# Comparative Analyses between Retained Introns and Constitutively Spliced Introns in *Arabidopsis thaliana* Using Random Forest and Support Vector Machine

**DOI:** 10.1371/journal.pone.0104049

**Published:** 2014-08-11

**Authors:** Rui Mao, Praveen Kumar Raj Kumar, Cheng Guo, Yang Zhang, Chun Liang

**Affiliations:** 1 College of Mechanical and Electronic Engineering, Northwest A&F University, Yangling, Shaanxi, China; 2 College of Information Engineering, Northwest A&F University, Yangling, Shaanxi, China; 3 Department of Biology, Miami University, Oxford, Ohio, United States of America; 4 Department of Computer Sciences and Software Engineering, Miami University, Oxford, Ohio, United States of America; University of California, Los Angeles, United States of America

## Abstract

One of the important modes of pre-mRNA post-transcriptional modification is alternative splicing. Alternative splicing allows creation of many distinct mature mRNA transcripts from a single gene by utilizing different splice sites. In plants like *Arabidopsis thaliana*, the most common type of alternative splicing is intron retention. Many studies in the past focus on positional distribution of retained introns (RIs) among different genic regions and their expression regulations, while little systematic classification of RIs from constitutively spliced introns (CSIs) has been conducted using machine learning approaches. We used random forest and support vector machine (SVM) with radial basis kernel function (RBF) to differentiate these two types of introns in *Arabidopsis*. By comparing coordinates of introns of all annotated mRNAs from TAIR10, we obtained our high-quality experimental data. To distinguish RIs from CSIs, We investigated the unique characteristics of RIs in comparison with CSIs and finally extracted 37 quantitative features: local and global nucleotide sequence features of introns, frequent motifs, the signal strength of splice sites, and the similarity between sequences of introns and their flanking regions. We demonstrated that our proposed feature extraction approach was more accurate in effectively classifying RIs from CSIs in comparison with other four approaches. The optimal penalty parameter C and the RBF kernel parameter 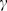 in SVM were set based on particle swarm optimization algorithm (PSOSVM). Our classification performance showed F-Measure of 80.8% (random forest) and 77.4% (PSOSVM). Not only the basic sequence features and positional distribution characteristics of RIs were obtained, but also putative regulatory motifs in intron splicing were predicted based on our feature extraction approach. Clearly, our study will facilitate a better understanding of underlying mechanisms involved in intron retention.

## Introduction

As an essential post-transcriptional process, alternative splicing (AS) can increase transcriptome plasticity and protein diversity [1]. There are primarily three types of AS: intron retention, exon skipping, and alternative choices of 5′ and 3′ splice sites (5′ss and 3′ss, respectively) of introns [2]. The frequency and types of AS differ significantly between vertebrates and invertebrates [3]. For example, only ∼19% of multi-exon genes are alternatively spliced in fruit fly, while it is ∼95% in human [4], [5]. In vertebrates and especially mammals, most alternatively spliced genes possess exons that are entirely spliced out or truncated, and intron retention is the least prevalent form of AS [6]–[8]. In invertebrates and plants, in contrast, more introns have their retention in mature mRNAs [3], [7], [9], [10]. A recent genome-wide study in *Arabidopsis* reports that ∼42% of the multi-exon genes undergo AS with ∼40% of those genes having retained introns (RIs) but only 3% having spliced exons [11]. Furthermore, it is likely that the number of AS genes identified in plants will keep increasing with the increased number of tissue-specific transcriptome studies. *Syed et al.* [12] reports that the AS events being found have risen from 1.2% to 61% over the past decade in *Arabidopsis*. Accumulating evidence indicates alternative splicing in invertebrates and plants might have different mechanisms in comparison with vertebrates and especially mammals, and the extent and complexity of intron retention in plants still need to be specifically characterized.

Transcript samples with RIs that are examined by RT-PCR are shown to co-purify with polyribosomes, suggesting that these intron retention events are not the result from incomplete splicing but are found in their nuclear exports [13]. Some researches show that specific abiotic stresses can impact on RIs. By analyzing the splicing process of a cold-regulated gene encoding ribokinase (7H8) protein, *Mastrangelo et al.* [14] suggests that 7H8 cold-dependent intron retention is a general trait in cereals. *Palusa et al.* [15] reports that various abiotic stresses affect the splicing pattern of serine/arginine-rich (SR) genes in *Arabidopsis*. On the other hand, there are many studies indicating that intron retention is a major AS phenomenon in plants [13], [16], [17], most of which concentrate on the positional distribution of RIs in 3′ UTR, 5′ UTR and CDS regions. However, it still lacks research on characterization, comparison and prediction of two types of introns using large amount of data by machine learning approaches in plants. Therefore, further works are required to deepen our understanding of RIs and unravel the underlying molecular and biological mechanisms.

Machine learning approaches have been widely applied to knowledge extraction from biological experimental data [18]. For classification of various problems in the domain of bioinformatics, prior studies suggest that SVM outperform k-nearest neighbors, neural networks and decision trees [19]–[21]. In SVM applications, the radial basis kernel function (RBF) that has only one kernel parameter 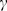 is widely adopted [22]. Unlike the linear kernel, it can handle data with nonlinear relations between class labels and features [23]. Only under certain parameters, the sigmoid kernel is valid and demonstrated to behave like RBF [24]. Additionally, the polynomial kernel has more kernel parameters and demands more training time than RBF, and it can easily fall into numerical difficulties with the degree increase [23]. Therefore, RBF is selected and used in our study. In the SVM training procedure with RBF kernel, both 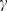 and the penalty parameter C settings are shown to significantly influence the classification accuracy [25]. Particle swarm optimization (PSO), a meta-heuristic optimization algorithm that simulates the social behavior of bird flocking or fish schooling [26], proves to be an appropriate approach in finding better parameters of SVM [27]. On the other hand, random forest has been reported as another competitive classification algorithm and received increasing interests [28], [29]. After surveys of random forest applications in bioinformatics for the recent decade, *Boulesteix et al.* [30] summarizes that random forest offers attractive features such as direct handling of high-dimensional data and advantages in parameters selection. Especially compared with SVM, it is easier for random forest to obtain excellent performance using the default parameterization without tuning parameters in general [31], [32]. Recent works show that random forest classifiers obtain better performance comparable to SVM in some bioinformatics applications including classification of cancer microarray data [33], identification of DNA-binding proteins [34], and prediction of miRNA targets [35].

Using random forest and in-house implemented PSOSVM that utilizes PSO to optimize parameters C and 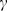 of SVM, our study was set up to detect systematically the differences between two types of introns, and characterize and categorize them accurately. Our proposed feature extraction approach is novel and hybrid, including three aspects: basic intron sequence features; frequent short linear sequence motifs; and features extracted from splice sites and the flanking sequences of introns. In our study, performances of random forest and PSOSVM to classify RIs and CSIs were analyzed and compared, and the results of classification based on different feature sets suggested that our feature extraction approach had a distinct advantage.

## Materials and Methods

### Dataset

RIs are defined if the introns are spliced out in at least one isoform (mRNA) but entirely retained in at least one other isoform for the same genes. In addition, for multiple RIs founded in different isoforms of the same genes, if the differences in the 5′ splice sites (or the 3′ splice sites) of these RIs are less than 6 bp, we define these RIs as redundant ones. Hence the longest one is selected among them for downstream data analysis. CSIs are defined as ones that are always spliced out in all isoforms of individual genes.

Based on TAIR10 gene annotation, coordinates of introns in genome sequences were determined using TAIR10_GFF3_genes.gff (ftp://ftp.arabidopsis.org/home/tair/Genes/TAIR10_genome_release/) by a Perl script. Then using GMAP [36], we extracted RIs and CSIs sequences, splice sites and flanking exons sequences of introns in *Arabidopsis* from the genome sequence files (ftp://ftp.arabidopsis.org/home/tair/Sequences/whole_chromosomes/). R *quantile()* function was employed to generate intron length quantiles for analyzing the intron length distribution in *Arabidopsis*.

### Feature extraction approach

Our new hybrid feature extraction approach combines the following three aspects:

#### (A) Basic features extraction

On one hand, we consider some of the most common global features of nucleotide sequences, such as intron length, nucleotide occurrence probabilities of A, C, G and T in introns, AT content and GC content. On the other hand, we determine local features of segmental nucleotides composition [37], which provide crucial complementary to the global features and are defined as **segmental probabilities of four nucleotides correlation factors** 
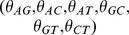
, as shown below:

For a 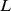-length nucleotide sequence (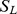):
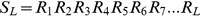
(1)

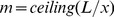
(2)


Here 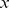 is set to 20 in our work, because the length of the shortest intron sequence is 20 bp in our datasets. 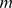 is the smallest integer not less than (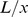).


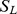
 is divided into 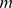 sections as following:
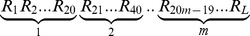



Each section includes 20 bp except the last section, which includes (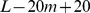) bp.
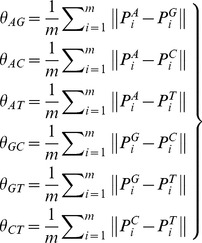
(3)


Here 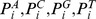 denote probabilities of the corresponding 4 bases (A, C, G, T) in the 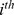 section respectively.

#### (B) Frequent motifs extraction

Because of the differences between RIs and CSIs, some subsequences appear more frequently in RIs than CSIs, or vice versa. In this paper, these motifs need to be more frequent in either RIs or CSIs but not frequently occur in both RIs and CSIs. We searched 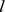-mer subsequences using sliding window with the step size of 1, and extracted all subsequences from 2 to 5-mer because 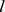-mer subsequences occur with low frequencies if 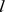 is greater than 5. For example, the mean frequency of 6-mer subsequences is low (2.01E-05). In order to discover frequent motifs from the above-mentioned 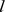-mer subsequences, evaluation indicators are required and defined as following:
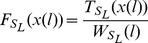
(4)

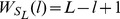
(5)


Here, 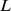 refer to the length of 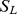 (Eq. 1), 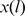 designates 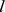-mer subsequence, 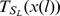 denotes the occurrence number of 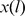 in 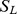 while 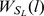 denotes the number of all 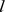-mer subsequences within 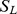. So 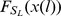 means the frequency of 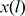 in 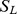, which will be the value of feature vector if 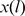 is determined as a frequent motif.
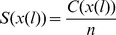
(6)


Dataset ({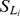}) include 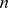 nucleotide sequences. In {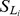}, 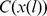 refers to the number of sequences in which 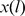 is discovered. 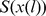 is used to describe the confidence of 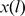 in {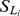}. In this paper, frequent motifs must have higher value of 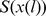 in either RIs or CSIs.
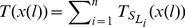
(7)

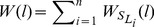
(8)

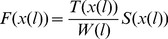
(9)



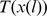
 denotes the occurrence number of 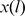 in {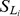}, and 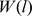 denotes the total number of 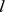-mer subsequence included in {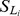}. 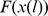 represents the frequency of 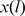 in {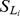}.
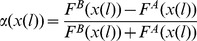
(10)


In Eq. 10, 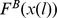 is the frequency of 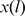 in dataset of CSIs, and 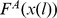 is the frequency of 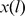 in dataset of RIs. 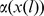 represents the relative difference of 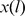 between CSIs and RIs datasets. The positive value of 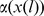 means a higher frequency of 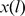 in CSIs than in RIs, the negative value of 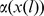 means the opposite case. So, we need to consider the value of 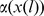 and 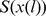 as a whole, and select appropriate thresholds of 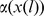 and 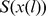 to decide frequent motifs.

#### (C) Splice sites and the flanking sequences of introns features extraction

**Figure 1:**
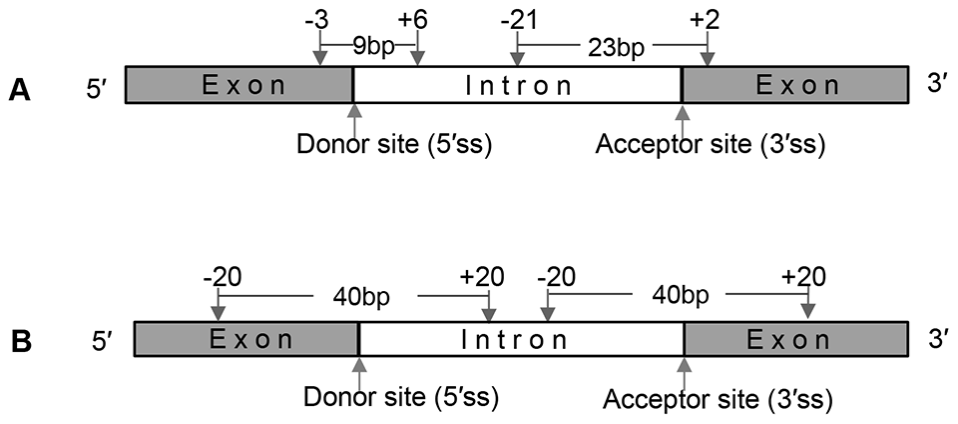
Feature extraction approaches for calculating signal strength of splice sites and similarity of intron and the flanking exons. A. The sequence extraction approach for calculating signal strength of splice sites; B. The sequence extraction approach for calculating increment of diversity (ID).

To quantify the signal strength of 5′ and 3′ splice sites, we extracted 9 bases for donor sites (−3∼+6) and 23 bases for acceptor sites (−21∼+2) from introns and their flanking exons (see details in Figure 1A), and then calculated frequencies of nucleotide A, C, G and T, which were selected as the parameters of position weight matrix (PWM) [38]. The PWM is defined as following:
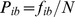
(11)

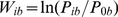
(12)


Here, 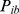 is the position probability matrix. 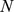 is the total number of sequences in the training sets. 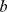 represents any of the four nucleotides: A, C, G, and T. 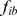 denotes the occurrence number of 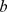 in the 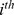 position of the 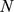 aligned sequences along the splice sites. 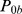 is equal to 0.25, and 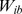 denotes the PWM value of 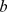 in the 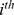 position. For a 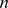-length sequence, the PWM scoring function (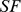) is defined as:
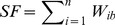
(13)



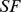
 denotes the quantitative value of the signal strength of splice site. The greater value of 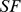 means the more probability of constructive splicing sites [39].

All of the sequences extracted from −20 to +20 bp at donor (acceptor) sites were separated into two datasets from splice sites (see details in Figure 1B): one exon sequences dataset and one intron sequences dataset. Increment of diversity (ID) is used to depict the similarity level of these two datasets [40]. The difference between RIs and their flanking sequences datasets (or CSIs and their flanking sequences datasets) can be quantitatively described by ID.

Let 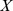 represents 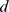-dimensional category space 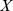: {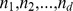}, the standard diversity measure for 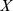 is defined as:
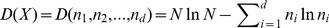
(14)


Here 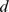 represents the total number of trimers, 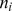 is the absolute frequency of the 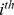 trimer in nucleotide sequence, 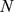 is equal to 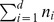. RIs have the similar trimer usage with the exons, which is different from CSIs where trimer frequencies are obviously different between introns and flanking exon regions [41].

For the two 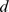-dimensional sources 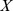: {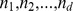} and 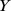: {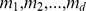}, ID depicts the similarity between the 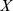 and 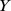. It is defined as:
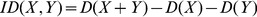
(15)


Here 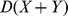 is the measure of diversity of the mixed source 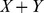: {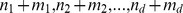}.

By the above-mentioned feature extraction approach, the sequence information in our dataset was changed into feature vector using R codes that utilize “seqinr” package (http://cran.r-project.org/web/packages/seqinr/index.html).

### Random Forest

Random forest is an ensemble classifier that consists of many independent decision trees [28]. Each tree is created by bootstrap samples of the original training data using a randomly selected subset of features [42]. At each split about 37% of the training data, named as “out of bag” (OOB) samples, is not used to construct but evaluate the performance of each classification tree [33]. The other remainder, named as “in-bag” samples, is used to construct each classification tree. Then individual trees are combined through a voting process to provide an unbiased prediction. Compared with other classification approaches such as decision tree, it possesses internal cross-validation [43] and could be more accurate and tolerant to noises [35]. The random forest algorithm is available in Weka [44].

### PSOSVM

SVM classifier, as a typical 2-class classifier, is to calculate an optimal linear separating plane that separates two classes of the dataset [45]. For non-linearly separable cases, samples are mapped into a high-dimensional feature space where a separating hyper plane can be found, and proper kernel function is sought to realize this nonlinear mapping [46].

In our study we used RBF kernel. Considering two samples 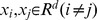, the RBF kernel is calculated using 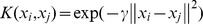, where 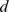 denotes the number of dimensions of input feature vector and 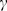 (>0) represents the width of RBF [47]. In general, the performance of SVM is determined by parameters (C, 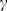). The grid search algorithm is a traditional method to find the best (C, 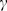) [48]. However, it is difficult to obtain a satisfactory outcome because of too limited parameter pairs to search from the huge size of possible search space by applying this method. *Lin et al.* [26] introduces PSO for parameter determination and feature selection of SVM, and experimental results demonstrate that the classification accuracy of SVM optimized by PSO performs better than many other parameter optimal approaches [49].

PSO consists of particles in the population that search for the best position by following its best solution [50]. A particle is considered as a point in a 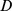-dimension space, and its status is represented based on its position and velocity. Let 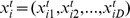 and 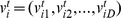 represent the 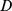-dimensional position and velocity of particle 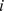 at iteration 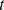 respectively. Let 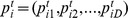 represents the best personal solution that particle 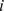 has obtained until iteration 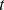, and 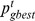 indicates the best global solution obtained from 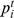 in the population at iteration 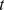. To search for the optimal solution, each particle updates its velocity and position as following:
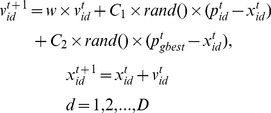
(16)


Here 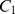 denotes the cognition learning factor, 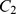 denotes the social learning factor, 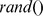 is positive random number which is uniformly sampled from the interval [0,1].

**Figure 2:**
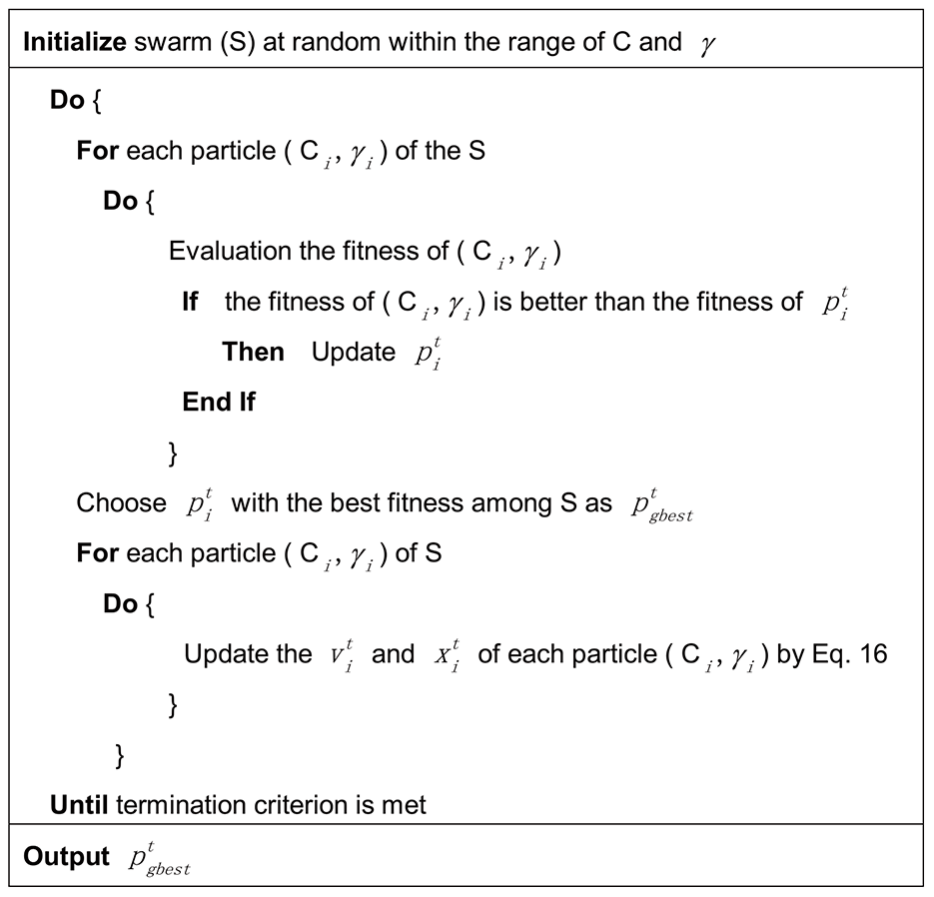
The pseudo-code of PSOSVM. The details of Eq. 16 are illustrated in Materials and Methods.

In this study, parameters of our proposed PSOSVM were set as shown in Table 1, and the pseudo-code of the PSOSVM was illustrated in Figure 2. We implemented PSOSVM algorithm in the eclipse platform integrated with Weka (http://www.cs.waikato.ac.nz/ml/weka/) and LibSVM (http://www.csie.ntu.edu.tw/~cjlin/libsvm). The program of our PSOSVM was written in java.

**Table 1:** The parameter values or ranges of PSOSVM.

Parameter	Value or Range
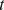 (the number of iterations)	10
S(the number of particles)	100
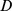 (dimensions of particle)	2
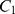	1.49618
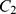	1.49618
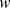	0.7298
C	(2^−8^, 2^10^)
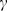	(2^−8^, 2^8^)

The rule-of-thumb settings of 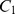, 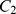 and 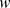 are cited from [74].

In order to select optimal parameters C and 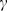 in the population, the fitness as an evaluation indicator in PSOSVM was necessary. Here the fitness of (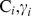) (Figure 2) was set to be the averaged accuracy of the SVM classifier on the training dataset via 10-fold cross-validation (10FCV) experiment.

### Performance assessment

Several assessment measures were used to evaluate the classification performance using random forest and PSOSVM in this study. All of them were deduced from the numbers of true positives (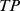), false positives (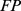), true negatives (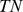) and false negatives (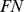) [51]:
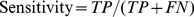
(17)

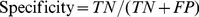
(18)

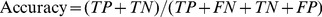
(19)

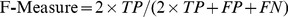
(20)


Here Accuracy (Eq. 19) represents the rate of overall correct classifications. F-Measure (Eq. 20) is often used as a single-value benchmark that characterizes classification performance. A receiver operating characteristics (ROC) curve plots True Positive Rate (i.e., Sensitivity) versus False Positive Rate (i.e., 1-Specificity) [52], providing a valuable tool to summarize the accuracy of predictions. The area under the ROC curve (AUC) is used to quantitatively compare the performances of different predictive models without regarding to class distribution or error costs. So we also evaluated the performance using AUC. Moreover, in our experimental data, we utilized probability estimates instead of −1/+1 class labels [53] for each test instance to generate more accurate ROC curve and AUC for PSOSVM.

## Results

### Experimental dataset

**Figure 3:**
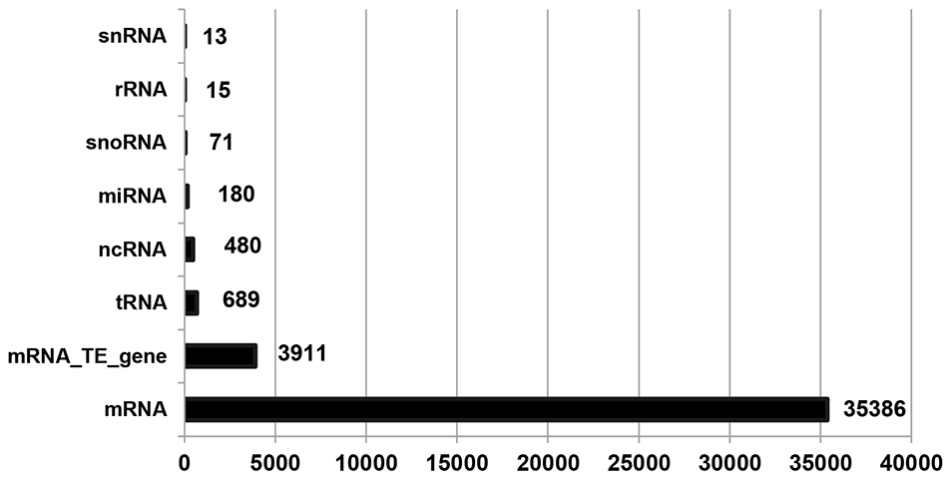
Numbers of various RNA types annotated in TAIR10 gene annotation for *Arabidopsis*. Each horizontal bar (with the number) indicates the number for a given RNA type.

In TAIR10 gene annotation for *Arabidopsis*, there are 28,775 genes, 3,903 transposable element genes and 924 pseudogenes. All these genes except pseudogenes have been used for further analysis, and they have a total of 40,745 annotated RNAs, which can be categorized into 8 different RNA types (Figure 3). It is clear from Figure 3 that most of the annotated RNAs are mRNAs (86.85%, 35,386 out of 40,745). As shown in Table 2, we found a total of 2,811 RIs and 113,098 CSIs in *Arabidopsis*. Interestingly, no RI was detected in chloroplast (ChrC) and mitochondrion (ChrM) while only 42 CSIs cases were found in these organelle genomes. For the 8 different RNA types, both RIs (98.26%, 2,762 out of 2,811) and CSIs (97.53%, 110,304 out of 113,098) were detected overwhelmingly in mRNAs whereas they (RIs: 1.74%, 49 out of 2,811 and CSIs: 2.47%, 2,794 out of 113,098) were rarely discovered among other 7 RNA types. Therefore, all the RIs (2,762) and CSIs (110,262 = 110,304-42) detected in mRNAs within chromosomes Chr1–Chr5 constituted our data set for downstream analysis.

**Table 2:** Distribution of RIs and CSIs in *Arabidopsis*.

Introns Categories	RIs	CSIs
All RNAs	2,811	113,098
mRNAs	2,762	110,304
ChrC, ChrM	0	42
Chr1, Chr2, Chr3, Chr4, Chr5	2,762	110,262
Redundant Cases	229	0

All RNAs means the 8 types of RNAs described in Figure 3. Redundant cases could only happen in RIs, the detailed description sees Materials and Methods.

Different from human genome that has much longer introns (5,500 bp in average) [54], *Arabidopsis* has much shorter introns. As shown in Table 3, the average lengths of RIs and CSIs are 145 bp and 160 bp respectively, and introns length varies greatly within a range from 8 to 10,234 bp. Based on the intron length distribution generated by *quantile()* in terms of the given probabilities (0.02, 0.2, 0.4, 0.6, 0.8, 0.98), 96% RIs and CSIs were found within the range from 44 to 501 bp and from 70 to 631 bp respectively. This suggested that extremely large introns (i.e., RIs: 2,075 bp and CSIs: 10,234 bp, 9,724 bp, 7,384 bp) and extremely small introns (i.e., those less than 20 bp) became outliers, which would cause a negative effect on classification. Consequently, we obtained the high-quality dataset including 2,520 RIs and 110,254 CSIs after removing these outliers (i.e., 13 RIs and 8 CSIs) and 229 redundant RIs (see the definition in Materials and Methods).

**Table 3:** Average size, range and sample qualtiles of RIs and CSIs.

Introns Categories	Average size (bp)	Range [Min,Max] (bp)	Quantile (bp)					
			0.02	0.2	0.4	0.6	0.8	0.98
RIs	145	[10–2,075]	44	81	92	112	182	501
CSIs	160	[8–10,234]	70	83	92	110	195	631

Quantile represents *quantile()* function in R. For given probabilities [0.02, 0.2, 0.4, 0.6, 0.8, 0.98], *quantile()* returns estimates of corresponding distribution quantiles based on sort order.

Supervised machine learning approaches for the identification of RIs and CSIs require a set of labeled samples [55]. In this study, RIs were regarded as positive samples and CSIs were regarded as negative samples. However, the proportion of positive to negative samples was approximately 1∶44, which was unbalanced and the performance of classification tended to be biased towards the negative class. To address this issue, under-sampling proves to be an efficient method for classifying unbalanced dataset [56]. We randomly selected three sets of 2600 CSIs from negative samples, by which we conducted our experiments and obtained similar results. So in this paper, we randomly chose one such set of 2,600 CSIs and integrated with 2,520 RIs as our final experimental dataset.

### A new hybrid feature extraction approach for classification between RIs and CSIs

As shown in Table 4, our hybrid feature extraction approach obtained 37 features (combining **A+B+C** features) for each intron in the experimental dataset. **A** denotes basic features, including both global features (e.g., Length, nucleotide occurrence probabilities of A, C, G and T, AT content, GC content) and local features (e.g., 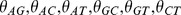). **B** denotes frequent motifs features, which are selected from all 2 to 5-mer motifs based on Eq. 4–Eq. 10, and have relatively high values of 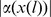 and 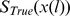 or 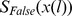. Among the selected frequent motifs, some of them (i.e., cc, gg, cg, ccg, cga, cgg, ggag, gggt, gaag, ttcg) have negative values of 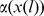 and higher values of 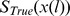. Whereas, others (i.e., ta, at, atgt, taat, tatat, atatt, aaata, ttata, attat) possess positive values of 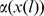 and higher values of 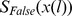. **C** denotes the signal strength features of the splice sites (SFvalue, SFaccvalue) and the similarity level features (IDdonv, IDacceptv) of two datasets, which include sequences from −20 to −1 and from +1 to +20 sites for 5′ and 3′ splice sites (Figure 1B).

**Table 4:** Feature vectors of experimental dataset.

Feature types	Feature vector
Basic Features **[A]**	Length; AT content; GC content; nucleotide occurrence probabilities of A, C, G and T; 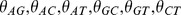
Frequent motifs features **[B]**	cc, gg, cg, ccg, cga, cgg, ggag, gggt, gaag, ttcg; ta, at, atgt, taat, tatat, atatt, aaata, ttata, attat
Splice sites and the flanking sequences features **[C]**	SFvalue, SFaccvalue; IDdonv, IDacceptv
Complete features [52]	Combined features (**A+B+C**) and 15 frequencies of trimmers (agg, ata, atg, cgc, cta, gcg, gga, ggg, gta, taa, tac, tag, tat, tcg, tta)
Optimized features [27]	Length, g, t, AT, 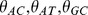 , cg, ta, cga, cta, gga, tac, tag, tta, gaag, ttcg, atgt, taat, attat, tatat, aaata, SFvalue, SFaccvalue, IDdonv, IDacceptv
Class label	True (RIs); False (CSIs)

Besides our hybrid feature extraction approach, we also built complete features (**52**) and optimized features (**27**) to classify RIs and CSIs (Table 4). All trimer sequences have more obvious differences between RIs and CSIs than dimers, and they also present higher frequencies of occurrence in our datasets than tetramers and pentamers. So we sorted values of 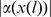 among all trimers and selected top 15 trimers with higher values of 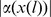. By integrating the frequencies of these 15 trimers with our combined **A+B+C** features, the complete features were obtained and defined as the **52** feature set. Moreover, we also employed the PSOSearch method to optimize the complete 52 feature set for getting better classification accuracy with less features. PSOSearch is a feature optimal selection method that implements the PSO algorithm. It is available in Weka 3.7.3. In the optimizing process of PSOSearch, the accuracy of random forest classifier was utilized to compare the classification performance of different feature sets. Finally, the optimized features were obtained and defined as the **27** feature set. The last feature is class label with True representing RIs and False representing CSIs.

### Evaluation of our hybrid feature extraction approach in comparison to other four feature sets

In this work, because of the diversity of different features (e.g., intron length, SFvalue and frequencies of frequent motifs), we firstly employed *scale* function to normalize values of individual features. Then, we selected 60% samples from the experimental dataset to verify the proposed feature extraction approach. Finally, the normalized feature vectors were adopted as inputs to classify RIs and CSIs by employing random forest and PSOSVM respectively.

By using PSO, the optimal parameters C and 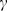 were selected and applied to test the performance of SVM classifier via 10-fold cross-validation. But for random forest, due to the “out-of-bag” error estimation, it is unnecessary to utilize cross-validation to obtain an unbiased estimate of the test set error [33]. We split 90% of samples for training whereas the remainder is used for testing the performance of random forest classifier. As shown in Table 5, the square root of the whole number of features is set for the parameter *numFeatures*, and the other parameter (*numTrees*) of random forest was set from 30 to 50 with a step size of 2 to find the optimal value using grid search algorithm.

**Table 5:** Optimal parameters and performances of random forest and PSOSVM using five different feature sets.

Algorithm	Feature set	Parameter (*numFeatures*)	Parameter (*numTrees*)	Accuracy	F-Measure	AUC
Random forest	**A**	4	42	0.771	0.772	0.867
	**A+C**	4	42	0.785	0.785	0.897
	Combined **A+B+C**	**6**	**42**	**0.808**	**0.808**	**0.900**
	Complete **52**	7	42	0.782	0.782	0.898
	Optimized **27**	5	42	0.788	0.788	0.891

Algorithm	Feature set	Parameter (C)	Parameter (*γ*)	Accuracy	F-Measure	AUC
PSOSVM	**A**	187.29	15.64	0.742	0.741	0.838
	**A+C**	1.0	5.5	0.771	0.771	0.842
	Combined **A+B+C**	**59.46**	**0.17**	**0.774**	**0.774**	**0.844**
	Complete **52**	1.40	0.22	0.771	0.771	0.843
	Optimized **27**	1.27	0.52	0.763	0.763	0.840

**Figure 4:**
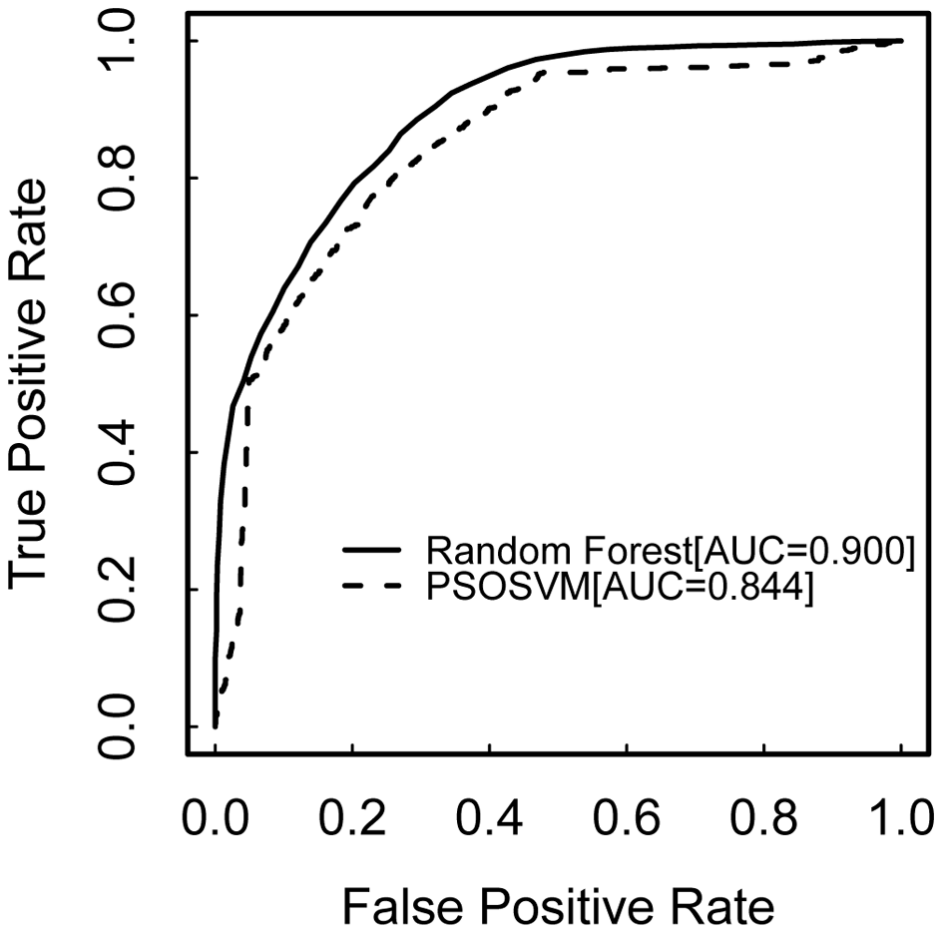
The ROC curves of random forest versus PSOSVM. The ROC curve of random forest is shown by the solid line and PSOSVM by the dashed line. The classification accuracy of these two methods is measured by AUC (the area under the ROC curve). Random forest gains significant advantages compared to PSOSVM (i.e., 0.900 versus 0.844).

In order to demonstrate the performance of our hybrid feature extraction approach, we employed five different feature sets to classify on our dataset: (1) **A** feature set, (2) **A+C** feature set, (3) our combined **A+B+C** feature set, (4) complete **52** feature set and (5) optimized **27** feature set (see Table 4). For each feature set, random forest and PSOSVM were carried out to do classification. The values of optimal parameters and performances of both two classifiers are shown in Table 5. Clearly, the combined **A+B+C** feature set showed better classification performances than other four feature sets for both random forest (i.e., Accuracy = 0.808, F-Measure = 0.808 and AUC = 0.900) and PSOSVM (Accuracy = 0.774, F-Measure = 0.774 and AUC = 0.844). On the other hand, based on these three assessment measures, the random forest classifier always achieved better classification performance than PSOSVM. The differential performance between these two classifier reached 0.056 obtained by AUC assessment measure using our combined feature set (Figure 4).

**Figure 5:**
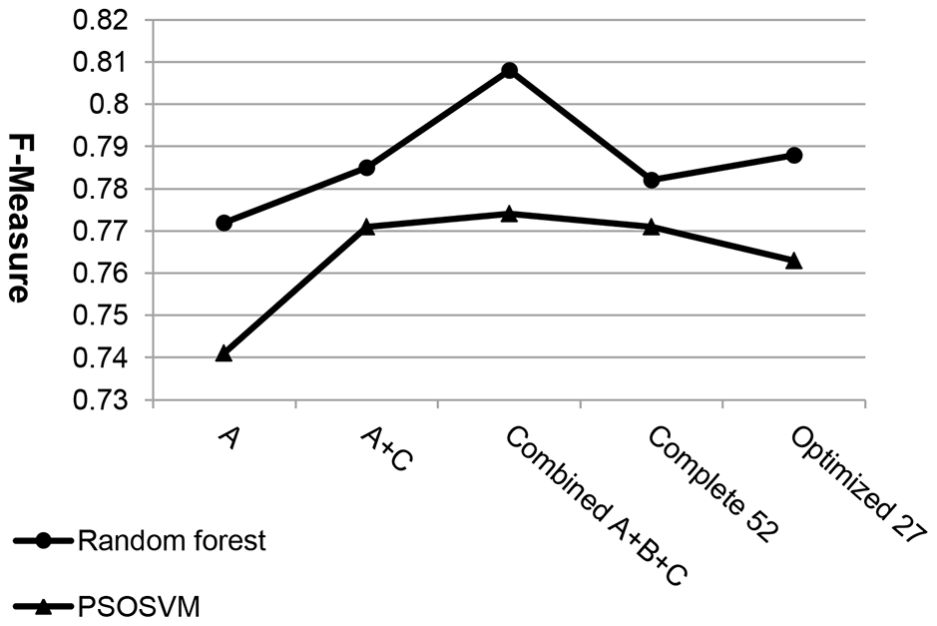
Performance of random forest and PSOSVM (F-Measure) in five different feature sets. Classification accuracy is assessed with F-Measure. Each solid round dot represents the accuracy of random forest and each triangle means the accuracy of PSOSVM for a given feature set. Compared with the other feature sets, our combined **A+B+C** feature set obtains the optimal classification performance by using both classifiers.

In Table 5, the performances of AUC appear to be better than those of Accuracy and F-Measure for all feature sets using random forest and PSOSVM. The performances of Accuracy and F-Measure are equal in all feature sets except **A**, in which the performance of F-Measure increases by 0.001 than that of Accuracy for random forest while the opposite case happens for PSOSVM. Interestingly, these five different feature sets display the same change trend of classification performance in terms of these three assessment measures for both random forest and PSOSVM. We focus on F-Measure (Figure 5) to illustrate this trend.

As shown in Figure 5, compared with our combined feature set, it is impossible to obtain better performance for applying sectional feature sets (e.g., **A** or **A+C**), complete **52** feature set, or optimized **27** feature set. This result suggests, not only for PSOSVM but also for random forest, that our hybrid feature extraction approach selected useful features for better classification between RIs and CSIs.

### The influences of short motifs, splice sites and flanking exon sequences in RIs

When we further investigated the influence of the feature sets in classifying RIs and CSIs, we discovered that **C** feature set made the greatest contribution to improve the classification performance: for example, 3% F-Measure increase using PSOSVM, and 3% AUC increase using random Forest for **A+C** feature set in comparison with **A** feature set (Table 5). As shown in Table 6, RIs have lower signal strength of splice sites (SFvalue = 3.930, SFaccvalue = 5.075) than CSIs (SFvalue = 4.806, SFaccvalue = 6.363). In addition, RIs have smaller values of IDdonv (17.934) and IDacceptv (17.891) than CSIs (IDdonv = 18.412, IDacceptv = 18.385), which suggests that intron sequences and flanking exon sequences for both donor sites (5′ splice sites) and acceptor sites (3′ splice sites, see Figure 1B) have higher similarity in RIs than in CSIs. The significant differences among these four features (SFvalue, SFaccvalue, IDdonv and IDacceptv) were detected between RIs and CSIs using one-way ANOVA (P<0.0001, see Table 6). This result demonstrated that these four features were indeed effective in classification between two kinds of introns.

**Table 6:** The mean value and P value of SFvalue, SFaccvalue, IDdonv and IDacceptv.

	SFvalue	SFaccvalue	IDdonv	IDacceptv
The mean value in RIs	3.930	5.075	17.934	17.891
The mean value in CSIs	4.806	6.363	18.412	18.385
P value (One–way ANOVA)	2.2e-16	2.2e-16	6.488e-07	3.545e-07

P value was calculated by applying F-test in one-way ANOVA based on experiment dataset included RIs and CSIs. The influences of classification among four features are all significant (p<0.0001).

**Figure 6:**
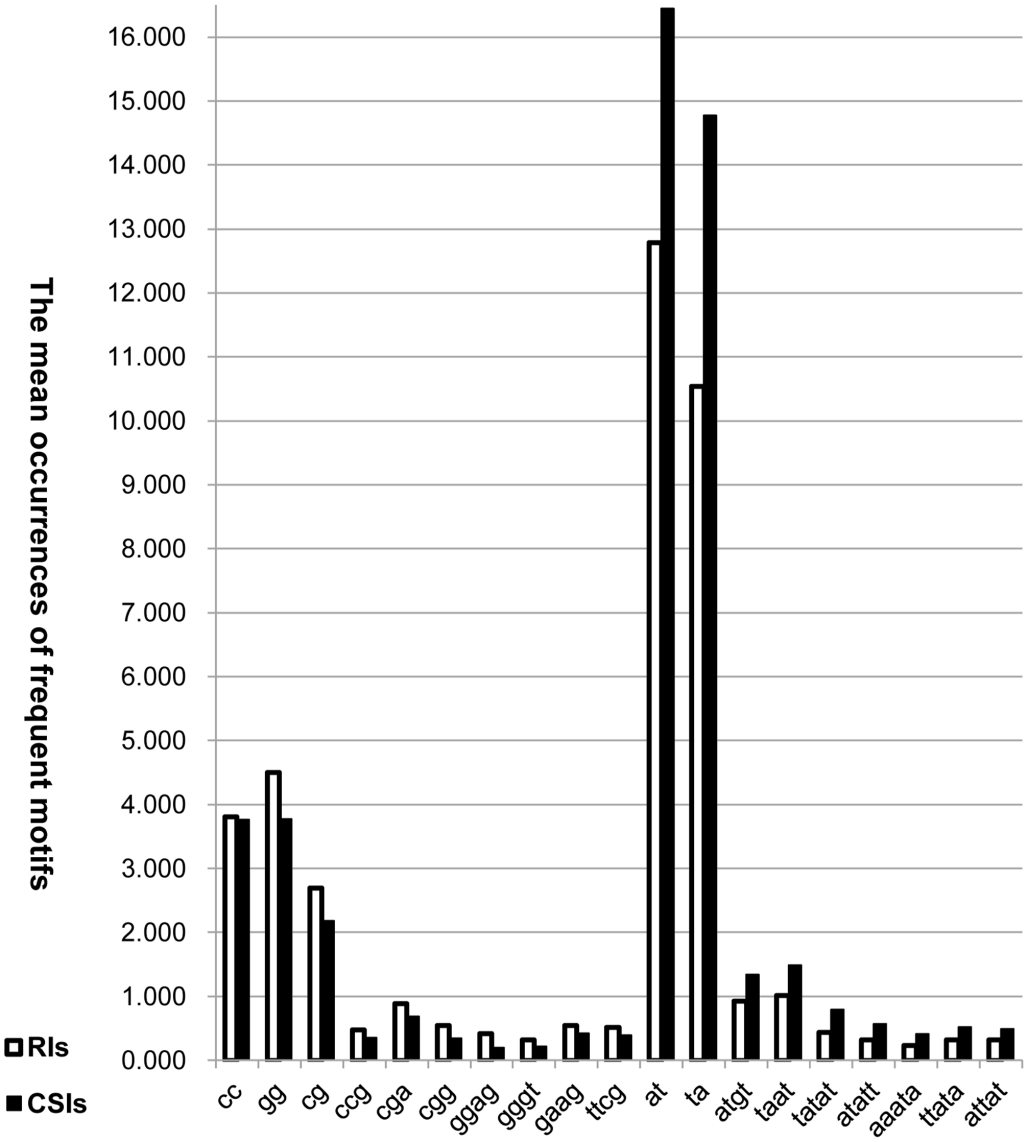
The mean occurrences of B frequent motifs between RIs and CSIs. In the left side of the histogram there are ten frequent motifs that have higher occurrences in RIs than in CSIs. In the right site of the histogram there are nine frequent motifs that have higher occurrences in CSIs than in RIs.

Meanwhile, we also found that some short motifs were relatively frequent but quite different between the RIs and CSIs. So we extracted **B** feature set, and the results showed that they indeed helped us improve the classification performance, especially by using random forest (e.g., 2.3% F-Measure and Accuracy increase for our **A+B+C** feature set in comparison with **A+C** feature set, see Table 5). As showed in Figure 6, some short motifs (e.g., cc, gg, cg, ccg, cga, cgg, ggag, gggt, gaag, ttcg) have higher frequencies in RIs than CSIs whereas others (e.g., ta, at, atgt, taat, tatat, atatt, aaata, ttata, attat) occur higher frequencies in CSIs than RIs.

## Discussion

Different from previous bioinformatics analyses of AS in *Arabidopsis* [11], [57], we used the most recent and well-annotated gene data from TAIR10 to extract our experimental intron dataset that consists of 2,520 RIs and 110,254 CSIs, and found RIs and CSIs showed distinctive characteristics in their sequences. We not only discovered similar features including shorter intron length, lower AT content and higher GC content in RIs with previous reports [13], [58], but also found 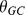 (14.3% versus 12.4%) was obviously higher and 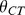 was conversely lower (23.0% versus 25.5%) in RIs than in CSIs. This indicates that difference between G and C contents for segmental intron sequences in RIs is greater than that in CSIs, whereas the difference between C and T contents for segmental intron sequences is higher in CSIs than that in RIs. As for the terminal dinucleotide splice signals of introns, there was no surprise that the consensus GT-AG introns (i.e., introns that begin with GT and end with AG) held 99% of CSIs and 96.7% of RIs. The second largest class, GC-AG introns, appeared more frequently in RIs than CSIs (2.61% versus 0.90%). This finding suggests that in *Arabidopsis* the unusual GC-AG introns appear to be more frequent in RIs than CSIs while the canonical GT-AG introns are richer in CSIs than RIs. Another interesting phenomenon in our data analysis is that more than half of RIs (58.4%) occurs in CDS, CDS+3′UTR or CDS+5′UTR regions. Such positional distribution characteristic of RIs indicates the potential that these introns are partly or entirely translated to proteins. Previous studies demonstrate that growing examples of cellular mRNAs with RIs express functional proteins by avoiding degradation through the nonsense-mediated decay (NMD) [59]–[61]. Our data analysis also provides a support for this trend by a high rate of RIs existing in coding regions.

It is well known that cis-acting sequences or motifs [62], such as enhancers and silencers in exons or introns, play significant roles for the regulation of AS. Plenty of studies indicate that exonic splicing enhancers and silences (ESEs and ESSs), most of which are known to bind SR proteins of the spliceosome, affect intron excision [63], [64]. *Pertea et al.* [65] has identified 84 putative exonic splicing enhancers (hexamers) in *Arabidopsis* by a computational approach. Although intronic splicing enhancers and silences (ISEs and ISSs) are less understood than ESEs and ESSs, a previous study also suggests [66] that these intronic splicing regulatory motifs also commonly impact on AS in mammals. Based on our feature extraction approach of **B** feature set, we analyzed all ggg-containing motifs with length from 3 to 5 bp included g triples (ggg, a well-established mammalian ISEs [67]), and found the mean value of 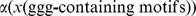 was −0.358, which indicated that ggg-containing motifs occurred more frequently in RIs than in CSIs. So the above result suggests these ggg-containing motifs, such as “gggt”, “gggtt” and “tgggt”, play a role of ISSs in *Arabidopsis*, instead of the role of ISEs in mammals. Of all ggg-containing motifs, “gggt” proves to contribute in distinguishing RIs from CSIs by our classification methods. In addition, the result of our extraction approach of **B** feature set also discovers that the mean value of 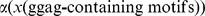 was −0.539, which indicated that ggag-containing motifs also have higher occurrences in RIs than in CSIs. In our study, the frequent motifs “ta, at, atgt, taat, tatat, atatt, aaata, ttata, attat” suggest some at/ta-rich motifs (i.e., ones include linear repeat or combination of “at” or “ta” (at least two “at” or “ta”)) may be ISEs in *Arabidopsis*. We checked all at/ta-rich motifs with length 4 and 5 bp and obtained the mean value of 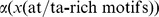 was 0.276, which illustrated at/ta-rich motifs had more frequently in CSIs than RIs. Furthermore, as the outstanding representatives of these at/ta-rich motifs, “taat”, “tatat”, “atatt”, “ttata” and “attat” have been proved to help recognizing the CSIs in our data analysis. Overall, ggg-containing and ggag-containing motifs seem to be ISSs because of their obvious abundance in RIs than in CSIs. On the other hand, at/ta-rich motifs appear to be ISEs because of their significant abundance in CSIs than RIs in *Arabidopsis*, which would potentially promote the identification of intronic splicing regulatory elements in plants.

Our results clearly demonstrate that random forest offers more advantageous classification performance than PSOSVM on five different feature sets. Performances of these two kinds of classifier are influenced by their respective parameters. Our experience showed that the parameter optimization was easier to implement for random forest (*numFeatures*  = 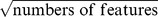, where *numTrees* is obtained by grid search), and the optimized parameters were beneficial to obtain stable classifier performance. In contrast, different values of (C, 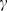) would cause large variation in the classifier performance of SVM [22]. Although we employed PSO to search the optimal parameters and have obtained better classification performance in comparison with the result using traditional grid search method, the classification performance of SVM may be further improved if the parameters could avoid trapping into local optima [68]. Unlike SVM, individual decision trees in random forest automatically utilize informative features more frequently in training process and achieve independent predictions, which were combined to gain accurate prediction of the forest [30], [69]. Therefore random forest presents significant superiority in failure tolerances and robustness, which plausibly explain the consistent advantageous performance of random forest classifier for all five feature sets in our study.

In this study, we utilized current TAIR10 mRNA (transcript or isoform) annotation in *Arabidopsis*, which does not provide any quantitative expression information (i.e., highly expressed versus rarely expressed mRNA) for alternate isoforms derived from the same genes. It is likely that highly expressed retained introns have different signal strength than retained introns with low expression levels. Therefore, utilizing RNA-Seq data to extract and incorporate expression information in intron level will definitely facilitate the development of more accurate and robust classifier by machine learning strategies. In fact, a recent RNA-Seq data analysis already shows evidence for novel transcripts and alternative splicing events in *Arabidopsis* that are not annotated in TAIR10 [70]. As more and more RNA-Seq and their meta-data (e.g., including environmental treatments, developmental stages and sampled tissues) are becoming available, more novel isoforms and previously un-annotated RIs will be evident in *Arabidopsis*, which can help us enhance the classification performance by providing more members within the RIs class. Moreover, we can do further classification of RIs that might be related to different environmental and/or developmental cues. Obviously, more RIs with different meta-data can be further analyzed to extract stress-, tissue-, or growth stage-specific features so that we can better understand how RIs are affected by both external and internal conditions in plants. On the other hand, RNA secondary structures have been demonstrated to affect alternative splicing [11], [71], [72]. Recently, the first *in vivo* genome-wide RNA structure map in *Arabidopsis* [73] highlights the importance of RNA secondary structures in alternative splicing (including intron retention). Therefore, a great challenge is how to accurately and effectively incorporate RNA secondary structures as features to enhance the performance and accuracy of our classifier. Without a doubt, a comprehensive feature extraction including both linear sequence features and RNA secondary structure features will definitely facilitate our understanding of how RIs are regulated in plants.

## Conclusions

The primary contribution of this work is our novel hybrid feature extraction approach that reveals overall features of introns, splice sites and flanking exons. These features can be utilized to effectively categorize and differentiate between RIs and CSIs. The experiments on five different feature sets verified that our combined **A+B+C** feature set could obtain the optimal classification performance by applying random forest and PSOSVM classifiers after tuning parameters. Follow-up analysis of these features has revealed interesting information about RIs in comparison with CSIs:

1. In average RIs have shorter length (145 bp versus 160 bp), higher GC content (35.76% versus 32.43%) and lower AT content (64.24% versus 67.57%) than CSIs.
2. RIs show different features of segmental nucleotides composition, such as higher 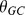 and lower 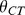 locally.
3. RIs possess lower signal strength of 5′ and 3′ splice sites (SFvalue, SFaccvalue), and terminal dinucleotide GC-AG appears a higher frequency in RIs than CSIs.
4. The RIs show higher similarity with their flanking exons than CSIs.
5. We here propose ggg-containing and ggag-containing motifs as ISSs as they are enriched in RIs. Accordingly, at/ta-rich motifs seem to be ISEs because of abundant in CSIs.

These features information about RIs can effectively facilitate an understanding of recognition mechanism of RIs in *Arabidopsis*.

## Supporting Information

File S1 Detailed introduction for how to extract data (File S2) using our source codes (File S3).
(DOCX)

File S2 All extracted data used in the article.
(ZIP)

File S3 All source codes used in the article.
(ZIP)
