# Supplementary material for: Comparative Analyses between Retained Introns and Constitutively Spliced Introns in Arabidopsis thaliana Using Random Forest and Support Vector Machine
Source: PLoS One. 2014 Aug 11;9(8):e104049. doi: 10.1371/journal.pone.0104049 (PMC4128822; doi:10.1371/journal.pone.0104049)
Supplement: File S1 — Detailed introduction for how to extract data (File S2) using our source codes (File S3). (DOCX) [file pone.0104049.s001.docx]

1. Environment and resources
2. Download TAIR10_GFF3_genes.gff from <ftp://ftp.arabidopsis.org/home/tair/Genes/TAIR10_genome_release/TAIR10_gff3>
3. Download genome sequence files (Arabidopsis) from <ftp://ftp.arabidopsis.org/home/tair/Sequences/whole_chromosomes/>
4. Build and install GMAP and GSNAP
5. Install R tools integrated with “seqinr” package
6. Install Weka (>=3.7.4)
7. Extract .fasta sequence files (File S1/extract_seq/)

Extract coordinates

RIs_coordinates.pl

CSIs_coordinates.pl

Filter Non-mRNA coordinates

Filter redundant RIs coordinates

Filter outliers coordinates

Get .fasta sequence files

filter_Non_mRNA_RIsandCSIs.pl

filter_redundant_RIs.pl (twice)

filter_outliers_RIsandCSIs.pl

get_RIsandCSIs_seq.pl

get_splicesites_seq.pl

get_IDdataset_seq.pl

1. Build our experimental dataset ----convert .fasta sequence into feature vectors (File S1/convert_feature_vec/)

2600 samples are selected randomly from CSIs_fasta_data

under_sampling_CSIs.R

A_features_RIs.R

Calculate A features

Calculate B features

Calculate C features

Build experimental dataset

A_features_CSIs.R

cal_S(x(l))_and_α(x(l)).R

C_features_RIs.R

expeimental_data.R

C_features_CSIs.R

B_featrues_CSIsandRIs.R

refvalue.csv

** 1) In refvalue.csv, ABfvalue means $\alpha(x(l))$, sxla and Bsxla mean$s_{True}(x(l)) \text{and} s_{False}(x(l))$. Based on the values of ABfvalue, sxla and Bsxla, cc, gg, cg, ccg, cga, cgg, ggag, gggt, gaag, ttcg, ta, at, atgt, taat, tatat, atatt, aaata, ttata, attat are selected as B features (frequent motifs).

2) Our original experimental dataset is allfeature.csv. Normalized feature vector dataset is ranscalefeall.csv. (File S2/rawfeatures)

3) Based on refvalue.csv, we also select top 15 trimers with higher values of $\left|\alpha(x(l))\right|$. Integrating them with our A+B+C features, the 52 feature set are obtained (**52_feature.R**) .

1. PSOSVM (File S1/ PSOSVM/)

Install eclipse integrated with Weka (<http://www.cs.waikato.ac.nz/ml/weka/>) and LibSVM (<http://www.csie.ntu.edu.tw/~cjlin/libsvm>)

1. Classify between RIs and CSIs using random forest and PSOSVM in Weka. (File S2/weka_data)
2. Convert .csv files to .arff files
3. Select 60% samples randomly from the experimental dataset to verify the accuracy of classification using random forest and PSOSVM. (feature5260.arff, featureA60.arff, featureAC60.arff, optimized_feature2760.arff, proposed_featureABC60.arff)
4. As shown in Figure 7, we employ PSOSearch and select random forest as attribute evaluator to optimize the 52 feature set. At last, optimized 27 feature set are obtained.
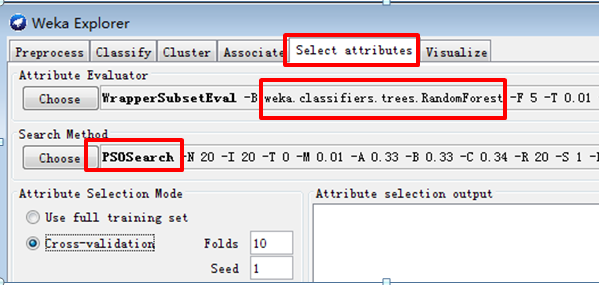


Figure7. The optimal implementation of the 52 feature set

1. Run “File S1/PSOSVM/TestPso.java” on feature5260.arff, featureA60.arff, featureAC60.arff, optimized_feature2760.arff, proposed_featureABC60.arff in turn and then obtain optimized parameters (Table 5), classify between RIs and CSIs using random forest and PSOSVM.
